# Artificial Intelligence Based Body Sensor Network Framework—Narrative Review: Proposing an End-to-End Framework using Wearable Sensors, Real-Time Location Systems and Artificial Intelligence/Machine Learning Algorithms for Data Collection, Data Mining and Knowledge Discovery in Sports and Healthcare

**DOI:** 10.1186/s40798-021-00372-0

**Published:** 2021-10-30

**Authors:** Ashwin A. Phatak, Franz-Georg Wieland, Kartik Vempala, Frederik Volkmar, Daniel Memmert

**Affiliations:** 1 Institute of Exercise Training and Sport Informatics, German Sports University, Cologne, Germany; 2 Institute of Physics, University of Freiburg, Freiburg im Breisgau, Germany; 3 Bloomberg LP, New York, USA

**Keywords:** Wireless body area networks, Wearable biosensors, Sports analysis, Real-time location system, Multi-sensor fusion, Vitals data

## Abstract

With the rising amount of data in the sports and health sectors, a plethora of applications using big data mining have become possible. Multiple frameworks have been proposed to mine, store, preprocess, and analyze physiological vitals data using artificial intelligence and machine learning algorithms. Comparatively, less research has been done to collect potentially high volume, high-quality ‘big data’ in an organized, time-synchronized, and holistic manner to solve similar problems in multiple fields. Although a large number of data collection devices exist in the form of sensors. They are either highly specialized, univariate and fragmented in nature or exist in a lab setting. The current study aims to propose artificial intelligence-based body sensor network framework (AIBSNF), a framework for strategic use of body sensor networks (BSN), which combines with real-time location system (RTLS) and wearable biosensors to collect multivariate, low noise, and high-fidelity data. This facilitates gathering of time-synchronized location and physiological vitals data, which allows artificial intelligence and machine learning (AI/ML)-based time series analysis. The study gives a brief overview of wearable sensor technology, RTLS, and provides use cases of AI/ML algorithms in the field of sensor fusion. The study also elaborates sample scenarios using a specific sensor network consisting of pressure sensors (insoles), accelerometers, gyroscopes, ECG, EMG, and RTLS position detectors for particular applications in the field of health care and sports. The AIBSNF may provide a solid blueprint for conducting research and development, forming a smooth end-to-end pipeline from data collection using BSN, RTLS and final stage analytics based on AI/ML algorithms.

## Key Points

- A large number of wearable sensor technologies have given rise to big data collection possibilities in the fields of sport and healthcare.
- Emergence of body sensor networks, real time location systems and multi sensor data fusion algorithm show great potential for application in wide set of industries.
- The proposed AIBSNF framework has potential to provide a solid blueprint for exploiting these rising technologies for end-to-end application from data collection to knowledge discovery across industries.

## Introduction

### Big Data and the Future

‘Dataism’ is a term coined by Yuval Harari in his popular science book ‘Homo Deus’. This term suggests that in the near future, decisions in all aspects of society will be based on the interpretation of the available ‘big data’ [1]. “Big data is defined as high-volume, high-velocity, high-variety and high veracity information assets (4Vs) that demand cost-effective, innovative forms of information processing for enhanced insight and decision making.” In this definition, volume refers to the magnitude or size of the data, variety refers to structural heterogeneity in the dataset, velocity refers to the rate at which data are generated and veracity refers to the truthfulness or reliability of the data [2–4]. ‘big data’ currently holds tremendous untapped potential, which has possible applications in a multitude of industries, including but not limited to health care, banking and finance, security, aviation, astronomy, agriculture, and sports [2, 5–7]. Although big data can be a considerable asset for the knowledge discovery process, using this data is a non-trivial task. Due to its unique computational and statistical challenges, the strength of ‘big data’ in terms of the 4Vs described above can also be its drawback. Noise accumulation, spurious correlation, measurement errors, and high computational power requirements are some of these challenges [8]. Solutions for any of the above-mentioned problems in terms of framework and data mining tools may prove crucial for generating data, analyzing it, and extracting actionable knowledge for application in the respective industries.

In recent years, the application of big data, its acquisition, and analysis using AI/ML algorithms have been applied in sports and healthcare diagnostics [5]. It has resulted in improvements in the identification of critical information and is being used in decision-making processes [5]. The nature of these fields is such that certain physiological signs that signify sports performance are also good indicators of mental and physical health [5]. The physiological information and movement patterns required to investigate athletic performance in sports, such as heart activity, recovery, muscular strength coordination, balance, etc., have considerable overlap with general health indicators. Considering this overlap, the data collection tools for these physiological indices can potentially be used to analyze both sports performance and available health predictors [9].

**Fig. 1:**
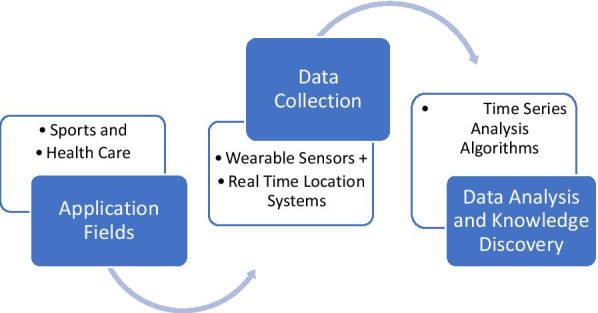
Application fields and Scope of the AIBSNF framework

Figure 1 outlines the scope of the present review, it outlines the field of application, technologies used for data collection and post data collection analysis for knowledge discovery in the broad set of fields.

### Status Quo of Data Gathering, Mining and Analytics in Sports

The use of big data and AI/ML tools in sports was first introduced in track and field, and weightlifting [10]. Baseball was one of the first sports to use data for recruiting and performance-enhancing purposes [6]. The sports of basketball and football soon caught up with a large number of professional teams and academics using ‘big data’ for recruiting, performance analysis, and performance enhancement in their respective sports [7]. The data currently collected in sports falls under broad categories, viz. physiological data, position tracking data, psychological data, scouting data, video data, etc. [7]. Spatiotemporal events, position data, and comprehensive match statistics for several sports are commercially available today through companies such as OPTA (https://www.optasports.com/), Hudl (https://www.hudl.com/), Instat (https://instatsport.com/), Statsbomb (https://statsbomb.com/), and others. Mining for the relevant information is primarily done through video and manual tagging [11]. The collection of physiological vitals such as muscular contraction data, GAIT analysis, etc., is currently difficult, if not impossible to extract just from the game footage. The quality of data available within the current technological limits is continuously improving, but some issues still persist in collecting high-quality data during live sporting events. Another issue is that data sources recorded by humans are currently prone to missing values, inconsistencies between different measures, and a temporal error [11]. Data sources that are automatically recorded, such as tracking information via motion capture systems, traditionally have difficulties with tracking complicated and crowded game situations [12]. Furthermore, video data's sheer size and complexity make it difficult for domain (sports) specific feature extraction [11]. This issue is sometimes dealt with manually or automatically after processing and cleaning the data; however, problems persist. Tracking a relatively small sporting object such as a ball traveling at high speeds is still unreliable even after post-event data mining [11]. Tracking high-speed objects such as balls and rackets is especially important since sporting equipment is usually the central reference point for meaningful sports analysis.

A recent review of sports research focusing on data mining and analytics (smart sports training) using AI/ML algorithms showed that the most researched sports involving big data and computational intelligence were soccer, running, and weight lifting [2]. The same review identified a total of 97 studies ranging from individual to team sports with the same focus. It also concluded that there is a lot of room for improvement in research methods with respect to the quality and public availability of datasets, which provides opportunities to validate the research done. Furthermore, multiple studies have implemented or proposed frameworks of ML algorithms and artificial neural networks (ANN) for sports results prediction. They mainly focus on technical implementation of the algorithms and its performance while predicting outcomes of a sporting match [2]. There seems to be less emphasis on frameworks that focus on obtaining and organizing the high-quality, low noise, and time-synced data required for implementing these algorithms [13]. The framework proposed in the current review aims to address these points.

### Data Gathering, Mining and Analytics in Health Care

The field of health care has a long history of recording, analyzing, and drawing inferences based on data [8]. This seems to be an effect of the requirements of regulatory bodies [8]. The total amount of healthcare data is predicted to cross Yottabyte scale in the next years [5]. Multiple approaches using AI have been used in the past for injury risk assessment and performance prediction [9]. Vison-based motion analysis has also been used for medical diagnostics[14]. There still seems to be untapped potential for big data to improve clinical operations, public health, preventive medicine, precision medicine, evidence-based medicine, remote monitoring, patient profiling, etc., in terms of lower cost, faster analysis, and reduced error rates [15]. A few of the factors crucial for accelerating innovation in the field of smart medicine seem to be, data gathering techniques and data mining from existing sources [16].

Architectural frameworks facilitating AI/ML have been proposed for analyzing the currently available vitals data across various sectors within healthcare [15]. Despite this, deep and smooth data integration across multiple healthcare applications is fragmented and slow. Another technical challenge for the development of such tools is the data from different healthcare environments. The lack of consistency in the structure of the data, available features, noise, and bias of the source may lead to issues in the trained algorithms [17]. With the development and advancements in low-cost near gold standard sensor technology, there is a possibility to combine multiple sensors to collect organized, time-synchronized data as the first step for developing a pipeline for use in numerous healthcare and diagnostics applications in the form of fusion technologies [18, 19]. Wearable sensors seem to be the ideal tool for collecting such high-quality data [20–22].

### Wearable Biosensors

The rise of wearable sensors as tools for data collection seems to be ideal for gathering physiological and vital data. Hence, wearable sensors have become popular in medical, entertainment, security, and commercial areas [21]. A recent review published in ‘Nature Biotechnology’ elaborated the rising interest in wearable biosensor technology in academics, performance, and the health industry [22]. Wearables show great potential to provide continuous, real-time physiological data using dynamic non-invasive measurements of biochemical and physiological markers. So far, these sensors have been used for gathering precise, high-fidelity strategic data, which facilitate a whole host of applications with, military, precision medicine, and the fitness industry at its forefront [23–25]. Their precise fidelity and precision varies based on the specifications of their varying use cases.

Advances in electronics, printing, non-invasive data collection, and monitoring technology, have given rise to durable, unobtrusive, non-invasive wearable clothing as an electronics platform capable of sensing human locomotion and vital physiological signals [21, 25–29]. Such media and miniaturization of sensors provide unprecedented capacity to gather a wide range of data in many scenarios. By choosing a specific set of sensors strategically located at different human body locations, there is potential to collect precise data for solving interesting problems. Table 1 shows a non-exhaustive list of non-invasive sensor technology that can potentially be used in various combinations to gather physiological data for applications in a wide range of disciplines.

**Table 1:** A non-exhaustive list of sensors and their applications in physiological vitals data gathering

Category	Physiological index	Application	Types of sensors	References
Sweat monitoring	Glucose and lactate	Blood sugar and physiological load monitoring	Iontophoresis electrode-based sensors	[20]
	Electrolyte concentration	Hydration monitoring, measuring of trace mineral densities, etc.	Galvanic skin resistance (GSR)	
Temperature sensors	Skin/core temperature measurement	Acute infection monitoring	Pyroelectric sensors	[30]
	Cortisol	Stress monitoring	Molybdenum disulfide nanosheets	[31]
Emotion regulation	Skin conduction	Continuous estimation of stress	GSR	[32]
Respiration	Oxygen saturation	Blood oxygen levels	Light emitting diode (LED)	[23, 33]
	Respiratory effort sensor		Strain sensor	
	Stretchable respiration sensors	Respiratory disease monitoring and diagnosis	Resistive humidity sensors	[28]
Cardiological data	Heart rate	Physical load	Electrocardiogram (ECG)	[34]
	Heart rate variability	Recovery and stress		
Skeletal muscle analysis	Electrical activity in muscles	Muscular abnormities, nervous system functioning	Electromyogram (EMG)	[35]
		Muscular fatigue, injury probability		
Continuous exercise monitoring through body motion analysis	Biomechanical data	Body position, postural control and team interaction	Accelerometers, pressure sensors, inertial measurement unit gyroscope	[36, 37]
	Foot movement pattern	GAIT analysis		

### Real-time Location Systems (RTLS) in Healthcare and Sports

*Real-time location system (RTLS)* is a combination of wireless hardware and software deployed to acquire a continuous real-time position of assets and resources, usually using a fixed reference point or receivers [38]. They seem to have an advantage over video capture as in most situations direct line of sight is required for motion analysis and this may not always be possible [39]. Most RTLS technologies are capable of measuring ToA (time of arrival), TDoA (time difference of arrival), AOA (angle of arrival), RSS (receiver signal strength), RSF (receiver signal phase), and RTF (roundtrip time of flight). Using this, they can identify the real-time location of an object(s) in question with one of the following methods: literation, angulation, or fingerprinting [40]. In recent years due to advancements in data collection technology, improvements in data mining algorithms and a reduction in the cost of development kits have given rise to a whole host of applications in industries such as production management, food delivery, healthcare and sports analytics [38, 41–44].

In healthcare, RTLS has been used effectively in elder care tracking, medical asset tracking, medication tracking, etc. Radio-frequency identification (RFID) has mainly been used in various capacities for improving medical asset management [34]. Furthermore, due to the COVID-19 pandemic of early 2020, RTLS was used for contact tracing to identify the potential spread of the virus. A combination of RTLS and electronic medical records was successfully able to locate all contacts with a sensitivity of 77.8% and specificity of 73.4%. Although not perfect, there seems to be potential to improve this rate by integrating other complementary measurement techniques [45].

In the field of team sports, real-time position and event data, in particular, has become crucial for the industry. Findings from this gathered data have benefited the field for physiological indicator analysis, tactical analysis, and their combination [46]. Despite considerable progress in motion analysis systems, there seems to be a lack of accurate and cost-effective technologies in the current market. The accuracy levels required for different marker-less human motion analysis scenarios are yet not established, but can potentially be improved by adding wearable tags (transmission antennas) on the players/sporting objects [46]. Multiple technology stacks and frameworks for data storage and potential analysis using AI and ML algorithms have been proposed [3]. Albeit, the gathering of such data in a synchronized manner is a non-trivial task. Multiple companies offer position data gathering and interpretation services using real-time location systems (RTLS) technology, but studies on the accuracy of this data are limited. Table 2 shows a non-exhaustive list of contemporary RTLS technologies, their accuracy, and detection range, in different applications.

**Table 2:** A non-exhaustive list of real-time location systems (RTLS) and applications thus far along with their specifications. (NLoS = no line of sight, LoS = line of sight,

Technology	Application	Dynamic accuracy	Detection range	Transmission frequency range	References
mmWave (5 g)	Human Pose detection	Up to < 0.02 m @ LoS	> 200 m@ outdoor	30–300 GHz	[47, 48]
Active radio-frequency identification (RFID)	Indoor location detection of devices and people	0.9 to 1.6 m@ NLoS	50–95 m radius with possible scalability to 1000 m @ outdoors	433	[42]
3D-Light Detection and Ranging (LiDAR)	Precision vehicle localization	0.01 to 0.2 m @LoS	~ 200 m @ LoS, both outdoor and indoor	~ 200 Thz	[49]
Wireless Fidelity (Wi-Fi)	Indoor and outdoor positioning for smartphones	1–3 m @NLoS	< 200, @ outdoor and < 60 m indoor under Wi-Fi covered distance	2.4 to 5 GHz	[42]
Ultra Sound	Indoor location	Up to 0.01 m @ LoS and ~ 0.02 @ NLoS	Up to 10 m @LoS indoor	1–20 MHz	[46, 50]
Bluetooth	Real-Time Indoor Positioning	Typically, between 2 and 5 m but can go up to 0.77 m using different signal processing algorithms	Up to 2 m @ NLoS	2 MHz of width in the 2.4 GHz band	[40, 51]
Ultra-Wide Band (UWB)	Tracking and position detection in sports	Between 0.08 and 0.2 m @ LoS	40–80 m	3.1 to 10.6 GHz	[42, 46, 52]
Computer Vision	Tracking of ball in sports such as Tennis and Cricket	Up to 0.05.–0.1 m @ 340 fps	N/A	N/A	[40, 53]
	Tracking path length of multiple objects	Up to 8.5% error and under 1 m for marker-based solutions	N/A	N/A	
Global Positioning System (GPS)	Measuring real-time movement of soccer players in a test situation	Up to 1.31 m/s error while measuring velocity and 6.05% error when measuring position @ NLoS	> 100 km Outdoor and Indoor	1575.42 MHz and 1227.6 MHz	[54]
Global Navigation Satellite System	Smartphone Location	Up to a few centimeters but unstable	> 100 km Outdoor	1–2 GHz	[55]

The fields of healthcare and sports have, to date, used RTLS and wearable technologies separately for solving specific problems [43, 45, 54]. The scope of the application has been limited thus far, but there seems to be a massive potential for using RTLS in combination with  wearable sensor technology. Previous research has proposed and implemented frameworks in health care and sports using these technologies separately. Still, there seems to be a lack of integration of these two technologies.

### Body Sensor Network (BSN)

Developments in wearable sensor technology and the improvements in wired and wireless communication devices have given rise to low-power, intelligent, miniaturized sensor node networks, also known as wireless body areas networks, body sensor network, body area networks, etc. (referred to as BSN henceforth) [56]. The BSNs provide a blueprint for placing sensors at strategic locations for each individualized application. BSNs have been proposed in a multi-level fusion framework (MLFF) to monitor soldiers and help decision-making by using multiple factors such as physiology, emotions, fatigue, environment, and location. The same MLFFs, which can potentially measure soldiers’ performance, can be used for sports performance analysis and health care with minor tweaks [24].

Biomechanical, biometric, and positional data are crucial for understanding physiology and logistics involved in sports. The data thus obtained, both in real-time and post-event, has tremendous potential in knowledge discovery, research and development across sports. This combination may help decode physiology and logistics in a sport, which may unlock further avenues for research and development in academics or applications in industry. Currently, several AI/ML algorithms exist which have the potential for performing multi-sensor data fusion. These algorithms can stitch images, perform time series analysis and forecasting, automatic event detection and classification, anomaly detection, fault detection, etc. [46–48].

### Artificial Intelligence and Machine Learning Algorithms for Multi-sensor Data Analysis

A comprehensive range of tools and techniques for time series analysis already exist for multidimensional signal processing. The utility has been demonstrated in applied and fundamental research in physics, biology, medicine, and economics [57]. A growing number of time series analysis algorithms have become available as data mining and interpretation tools due to recent advances in AI and ML. They seem to have an excellent tool capable of handling multivariate data. Table 3 shows a non-exhaustive list of algorithms used for multiple analogous applications that have the potential to be used in the problems addressed by the current review.

**Table 3:** Non-exhaustive List of Algorithms used for multi-sensor data modelling

Algorithm	Task	Performance	Category	References
Kernel Ensemble Random Forest classifier with 40 estimators, 8 features at depth of 15	Heart disease prediction using daily activity data from multiple sensors	98% accuracy on testing data	Medical Data	[18]
Convolutional Neural Networks	Fault diagnosis in a planetary gearbox from multi-sensor data	93% to 99% accuracy on testing data	Machine Design	[58]
Long Short-Term Memory Artificial Neural Network	Real-time identification of foot contact and foot off by analyzing gait pattern in children	~ 95% with maximum delay of 3 s in real time	Human Motion Analysis	[59]
TimeNet Pre-trained Deep Recurrent Neural Network	Generalized time series classification across multiple datasets	The average accuracy observed was 83% on various datasets	Generalized solution for series analysis across various domains	[60]
Choquet Integral + Hidden Markov Chain Models	Multivariate Time Series Anomaly Detection across various data sets	Between 90 to 99% depending on the chosen dataset	Anomaly detection	[61]
Convolutional Neural Networks	Real-Time Skeletal Posture estimation using mm-wave radar	Localization error of 3.2 cm for X, 2.7 for y and 7.5 for z	Human Motion Capture	[39]
Principle Component Analysis + Toeplitz Inverse-Covariance Clustering	Multivariate Time series analysis for identification of recurring events in smart manufacturing	Performs best across multiple performance matrices (F1, Precision, Rand Index, etc.)	Automatic Event Detection	[62]
K-nearest neighbors	Method for Recognition of the Physical Activity of Human Being Using a Wearable Accelerometer	78.9% accuracy	Activity Recognition	[63]
Support Vector Machines	Fall detection on mobile phones using features from a five-phase mode	Recall 90% and precision 95.7%	Activity Recognition /Fall detection	[64]
Artificial neural networks	An alternative to traditional fall detection methods	Sensitivity 0.984 Specificity 0.986	Activity Recognition /Fall detection	[65]
Bayesian sequential analysis and Multilayer Perceptron	Contamination Event Detection with Multivariate Time-Series Data in Agricultural Water Monitoring	Average detection rate > 80%	Water Contamination Event Detection	[66]
Fisher's Linear Discriminant	Detecting Stress During Real-World Driving Tasks Using Physiological Sensors	Accuracy of 97.4%	Stress Level Detection	[67]
Correlation-based feature selection with random forest classifier with random forest classifier	Automated epileptic seizure detection	Average accuracy of 98.45%	Medical Diagnosis	[68]

A large number of algorithms are continuously being developed in the field of AI/ML-based time series analysis. Multiple libraries in Python and R exist along with open-source repositories on GitHub. There are various tools available for analyzing and interpreting multivariate data acquired from multiple sensors [18, 19, 58]. However, multiple sensors in different fields seem to be univariate or exist only as theoretical frameworks. There appears to be limited applications of using continuous, time-synchronized physiological vitals and RTLS data in combination with AI/ML algorithms both, in sports and healthcare.

The present review aims to highlight available tools in fields of wearable biosensors, RTLS, and AI/ML. Furthermore, the authors propose, AIBSNF, a framework of BSN which collects continuous multivariate physiological and live location data through the mashup of RTLS and wearable sensor technology in a potentially time synchronized manner. AIBSNF provides the blueprint for collecting such data, which is ideal for knowledge discovery through AI/ML algorithms. The authors of the current review highlight the framework’s application in two widely distinct scenarios, viz. team sports (tackle in football) and healthcare diagnostics (monitoring and research in patients with rheumatoid arthritis or osteoarthritis).

## Artificial Intelligence-based Body Sensor Network Framework: AIBSNF

**Fig. 2:**
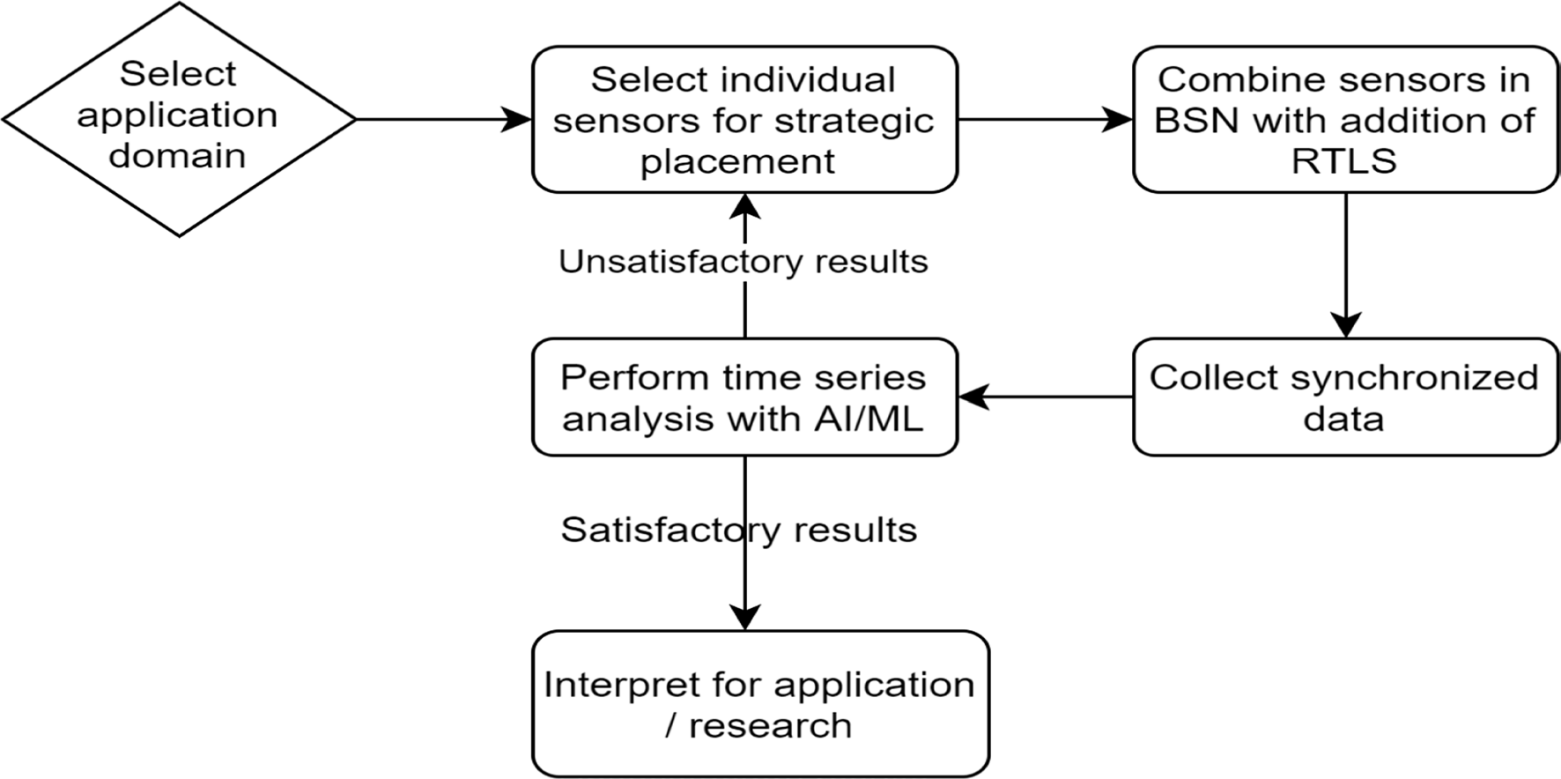
The conceptual framework (AIBSNF) for building a knowledge discovery pipeline

The selection of the sensors and placement on the body is crucial due to the limitations on the number of sensors placed on a single person. Furthermore, the sampling rate for different sensors may be different, which needs to be considered depending on the applications at hand. In Table 4, we suggest an example BSN framework and strategic placement of selected sensors based on successful past applications and fit into the conceptual framework shown in Fig. 2.

**Table 4:** List of chosen sensors and their possible placement based on previous applications and prior studies conducted

Chosen Sensor	Measurement Of	Placement on the body	Successful past applications	References
Inertial Measurement Unit (IMU)	Acceleration of the limbs (Angular and Linear)	Centre of Mass, Wrists, Feet (as Insoles)	Clinical instrumentation, falls management, identification of pathologic motor features, etc.	[69]
			Injury prevention, load assessment, performance coaching tool, automatic event detection in multiple sports, etc.	[10]
Electrocardiogram (ECG)	Detailed electrical activity of the heart, Heart Rate, Heart Rate Variability, etc.	On the chest	Abnormal Findings in ST and T waves in patients with Rheumatoid Arthritis	[70]
			Detection of unusual heart electric field parameters in type 1 and 2 diabetic patients	[71]
			Differentiating pathological vs physiological abnormalities in athletes to assess vulnerability of athletes to Sudden Cardiac Death	[72]
Electromyograph (EMG)	Electrical activity in the muscle in question	Quadriceps, Glutes, Calves, Hamstrings, Back, Abdomen, etc. (can be variable based on the problem at hand)	Assessing muscle activity levels in the elderly, patients with neurological disorders, and the injured	[69]
			Injury recovery, muscle activation patterns analysis, synergies in muscle chains, in athletes during sport specific movements	[10]
RTLS tag	Position of the person in question, from a set reference point	Center of Mass	Tracking of patients, medical staff and medical assets in a hospital	[73]
			Physical Load, real-time position data acquisition, tactical analysis in team sports, coaching and strategy development	[7, 74]
Force Plate	Pressure distribution on individual feet	Feet (as Insoles)	Gait, biofeedback interventions in stroke patients to improve balance, mobility	[69]
			Gait, analysis of athletes for technique and performance optimization	[10]

The accuracy of the abovementioned sensors is highly dynamic and the state of the art is constantly changing due to improvements in technology and post collection signal processing techniques

**Fig. 3:**
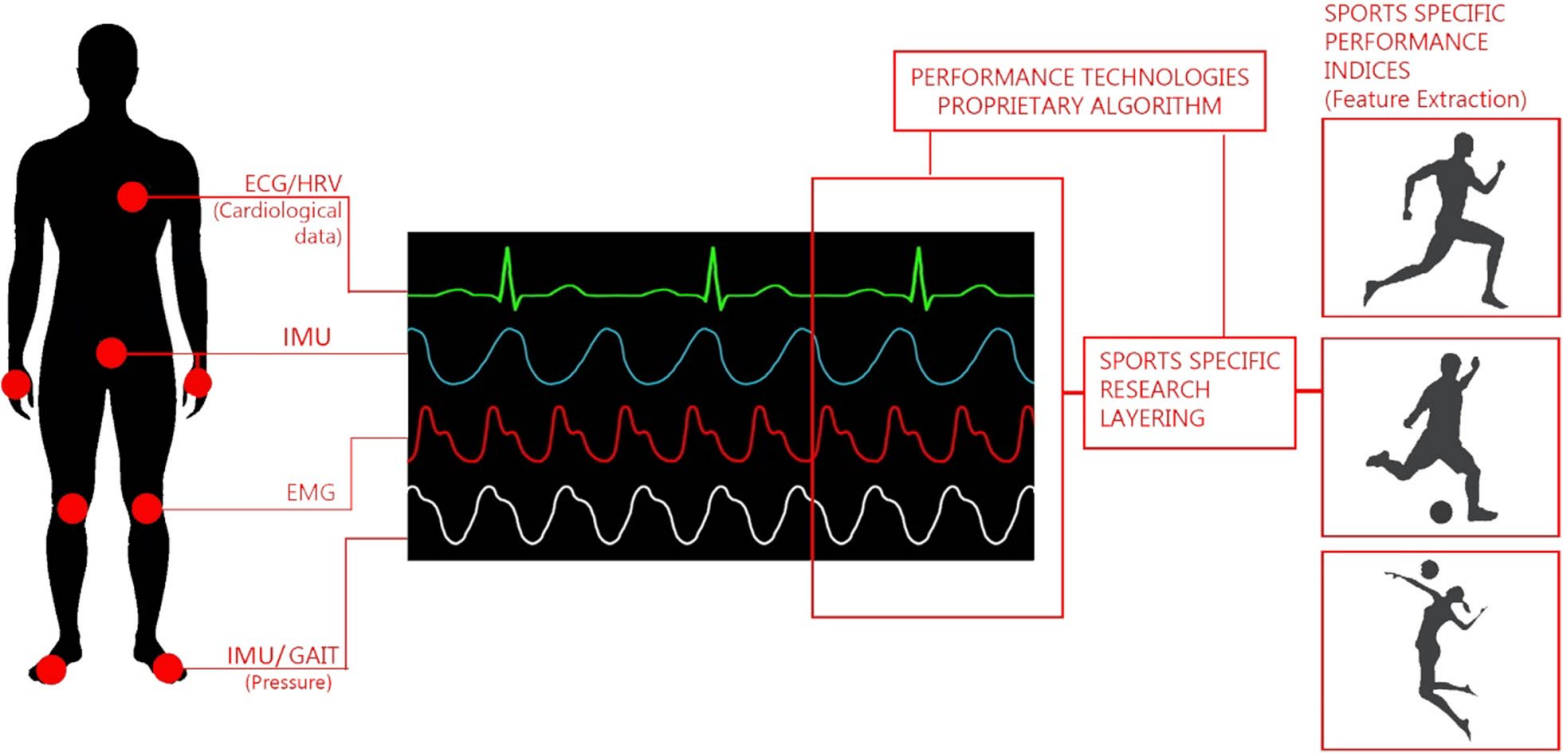
Example framework with selected BSN for continuous monitoring of chronic diseases and sports specific event detection

Figure 3 illustrates the whole proposed framework by using sample sensors, their ideal placement, data gathering and preprocessing as synchronizing the data, use of time series analysis algorithms for specific used case of sport specific event detection.

## Specific Applications

### Monitoring and Research in Patients with Rheumatoid Arthritis or Osteoarthritis

Rheumatoid arthritis (RA) is a chronic inflammatory autoimmune disorder that affects the joints but can also cause damage to other systems such as skin and lungs. It is projected that over 78 million adults will be diagnosed with rheumatoid arthritis in the US alone. Among those with arthritis, one in four have movement and working limitations. Furthermore, adults with arthritis were shown to be 2.5 times likely to have fallen as compared to healthy adults (https://www.cdc.gov/arthritis/data_statistics/arthritis-related).

Multiple studies have explored compliance rates as compared to the gold standard while performing rehabilitation exercises in patients with knee osteoarthritis. This was done using IMU sensors for feedback and monitoring resulting in varying degrees of accuracy during measurements. The studies concluded that wearable technology for assessing rehabilitation performance is a viable solution with room for potential improvements in measurement accuracy and compliance with the gold standard [75, 76]. Furthermore, ECG abnormalities have been detected in patients with RA, and there is a need to explore this area further [70].

AIBSNF can be used to continuously monitor individuals with arthritis. According to previous research there are observed reductions in stride length, EMG activation of specific muscles, and abnormalities in the ECG pattern (all known markers of progression of arthritis) [70, 71]. Interventions could be potentially timed to manage attrition in RA patients using medical or nonmedical information, such as exercise prescription and change in medications. Furthermore, the data collected from such continuous monitoring can be used by doctors, researchers and other health professionals to measure the efficacy and compliance rates of the intervention.

### Example: Automatic Detection of a Tackle in the Game of Football

**Fig. 4:**
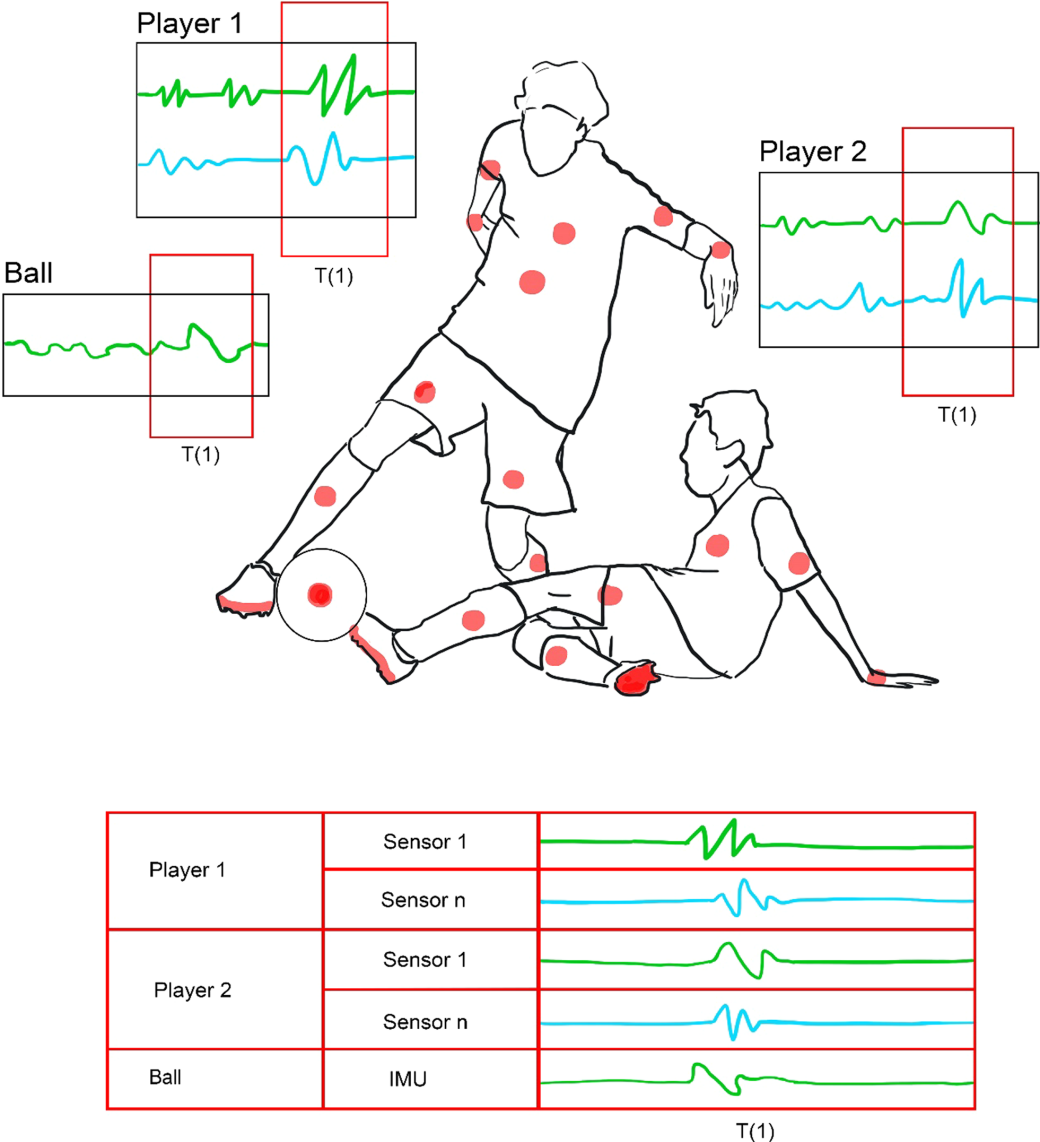
The scenario of conducting a tackle in the sport of football where both the players are fitted with a body sensor network

With an increasing number of teams taking decisions based on data analytics, there is a requirement for gathering and interpreting many high-fidelity data in sports specific scenarios. Automatic event detection and accurate position data are both crucial for this purpose. Figure 4 outlines a procedure in which ECG, EMG, gyroscopes, and RTLS tags have been placed at strategic locations on players from opposing teams and the ball. All these sensors would collect real-time data which convey the biomechanical and vital information from players, as well as the ball’s position. The scenario in Fig. 4 is a tackle which has a unique visual fingerprint, i.e., where the player without the ball (player 2) is on the ground with one leg extended, trying to reach the ball. Player 1 is trying to dribble  trying to reach the ball, which is in the possession of player 1.

Human experts can identify a tackle when they see it. This is primarily due to the physical interaction of two players, which is unique to the tackle itself. When this information is digitized using the BSN, the physics of the ball and biomechanics, combined with the location data of all three parties involved can be recorded. This data can be used for automatic sports-specific event and lower limb movement detection [77, 78]. A time-series clustering and classification algorithm can potentially identify all sets of tackles automatically. Another advantage of tracking ECG, EMG, and RTLS data is tracking of physical load on the cardiovascular system and individual muscles can also be performed. When done on an ongoing basis, there is potential to avoid injury, assess the preparedness of an athlete, and find new correlations in a host of technical and tactical components of the game in real-time [7, 74].

The same methodology can potentially be applied across multiple individual and team sports to identify a wide variety of events. Automatic event identification has been proposed in previous studies, but there is further research warranted due to low reliability and validity of existing approaches [37, 77]. The current BSN plus time series analysis framework would potentially prove invaluable for multiple applications in sports, including but not limited to analyzing technique, coaching, self and opponent analysis, tactical analysis, talent identification, player selection, recruitment etc. [7, 74, 79]. Furthermore, broadcasting agencies can use such data to provide visualizations and real-time information breakdown for live sporting events. This may help enrich the ordinary viewer's experience, providing them with in-depth information from an expert’s perspective. Health performance tracking is another up and coming field due to the rise of high-quality low-cost sensors. AIBSNF can be potentially used to build biofeedback mechanisms for continuous health tracking for a whole host of applications such as sleep and recovery tracking, personalized training programs for strength, mobility, cardiovascular endurance, and even ergonomic posture feedback in workplaces [56].

## General Applications

### Smartphone Applications and Wearables in Fitness and Health Tracking

Due to properties such as utility, portability, high computing power, the smartphone has become an ideal tool for collecting a wide variety of data including health-related parameters in everyday life [64]. Using technologies such as WiFi and bluetooth, these devices provide a perfect ground to stack systems of data transmission from a wide range of sensors in a simplified manner. The collection of data can be achieved by the smartphone itself with embedded sensors (e.g., triaxial accelerometer) or with the use of external sensors (e.g., ECG, EMG) using wearables [80]. Furthermore, the smartphone can potentially  act as a data transmitting and processing tool  that collects data and interacts with it. These properties facilitating  input, output and interactive operations make the smartphone an important part of the data management systems [21]. Various smartphone applications  combine data transmitted from different sensors which can be analyzed both in real-time and post collection [81].

Mashup data collection tools have  been applied in several studies such as the “Physiodroid study” [82]. Using external sensors and a smartphone for collection and computation, the team simultaneously analyzed ECG, heart rate, respiration, acceleration and skin temperature. Data was transmitted into an app where the patients  and the clinicians had insights into the processed data. Such data can prove useful to detect emergency events and have  shown to reduce anxiety in patients [82].

Besides the medical sector, ordinary users can profit from such integrated systems when applied in  individual sports. Connecting data on e.g., muscle oxygen levels  with heart rate there is potential for having  high fidelity view on the condition of the body during  training sessions, which could be useful for managing physical load and intensity on a personalized level.

### Applications in Healthcare Diagnostics and Research

A set of clinical case studies performed with patients on the autism spectrum compared the use of multivariate and univariate data using ML methods, to demonstrate the utility of multivariate analysis. The authors concluded that multivariate analysis techniques seem crucial for analyzing data collected from biological networks [83]. Furthermore, most diseases seem to have multiple causes and prognosis which are usually determined by several factors. The multivariable analysis allows accounting for the multifaceted nature of risk, and their relative contribution to the result. Hence it seems to be advantageous to gather multivariable data for diagnosis and designing probable interventions [84]. In line with previous research the proposed AIBSNF framework can be integrated in textile-based wearables [29]. These can potentially be self-powered, e.g. using recent advances in piezoelectric technology by harvesting energy from biomechanical movement [27, 85]. The wearables can also be extended to smart prosthetics and other assistive technologies [86].

### Applications in Alternative Medicine

‘Pulse diagnosis’ is a diagnostic technique used in Ayurveda, traditional chinese medicine (TCM), and other alternative therapies. Practitioners of these alternative medicine fields understand the pathological changes in internal organs [87, 88]. Devices capable of quantifying pulse via multi-sensor information using ECG, ultrasound imagining, pressure impedance blood flow, and volume pulse have been developed [89]. Furthermore, pulse diagnosis has also been conducted by measuring skin impedance at acupoints using a photoplethysmography sensor, galvanic skin response (GSR), and a smartphone. This was done to diagnose a condition called wiry pulse  in TCM with an accuracy of above 90 percent [90]. Research in areas that combine modern technologies to quantify and validate ancient medicine practices, seem to require multivariate vitals data and its analysis. AIBSNF with slight modifications may prove valuable for conducting validation studies and develop alternative medicine technology.

### Playing Field for Artificial Intelligence and Machine Learning

Chess has been used as an ideal scenario to push AI/ML research forward. This is because each piece has a strict set of rules as to where it may move. Furthermore, performance in chess is highly objective due to the strict set of rules. This allows the digitization of all moves performed, which can then be studied using probabilistic mathematical modeling. This makes it ideal for training agents via reinforcement learning [91]. Using the proposed BSN and RTLS technology, real world sports can be digitized in the same manner. However, the players in sports don’t follow rules as strict as those in chess. Sports are still largely contested under set of rules which are liberal as compared to chess. This, when digitized, can potentially provide a vast playground for AI/ML algorithms. This may offer a multitude of insights into specific sports and may also help move the field of reinforcement learning forward.

## Limitations and Issues

Although there is enormous potential for the use of BSNs, several challenges still exist for its implementation. On the technology side, the wearable sensors developed are comparatively new and collect data at different frequencies. They also lack benchmarking and approval for use in the medical industry [21]. The energy required for various sensors is varied, and there exist limitations such as data collection time due to the size and the shape of the battery, compliance of the user, and the physical impact of sensor operations. Hence, designing effective BSNs for solving problems requires a comprehensive set of domain-specific and technological knowledge [92]. Furthermore, on the analytics side, there still exist challenges such as computational power availability, high dimensionality, noise, and variable latency from different sensors in the case of real-time data collection [21]. The field of AI/ML, although advanced, is still in its infancy for discovering causal relationships. Hence, care must be taken to interpret output from AIBSNF as causal inferences or diagnosis, in the case of healthcare applications.

Data security is another major issue in the development and use of AIBSNF. Due to the intimate nature of the collected data, there needs to be mechanisms ensuring strict data protection at all proposed pipeline levels [56, 92]. The BSN connected to a user and data storage location may be significantly threatened by outside attacks. Data security risks may be higher for attacks from some entity with inside access to this data [22]. These issues are not unique to the BSN framework but for big data in general. However, due to the nature of the data collected in the applications mentioned in the current review, the threat may be amplified in terms of confidentiality, integrity, and information privacy [93].

In combination with data, using AI algorithms as independent decision making tools is a matter of great debate in the healthcare sector due to privacy concerns [94]. In the context of healthcare, AI can be categorized in two different ways, the first being a diagnostic tool, where the decision making and ethics lie with the human user of the tool. The second category is AI algorithms as independent decision makers or non-biological healthcare professionals [94]. The scope of the current paper lies within the prior category. A recent review outlines the opportunities and drawbacks of using AI in personalized medicine. It concludes the necessity for a multidisciplinary, public discussion to define the principles, ethics and social guidelines for using AI in healthcare due to limited expertise of the regulation sector in the field of concern [95].

## Conclusion

The fields of RTLS and wearable biosensors are rapidly developing. There has been tremendous progress in improving accuracy, validity, and reliability in the sport and healthcare industries over the past decade [22, 40]. It is safe to assume that they will continue to improve with the further layering of AI/ML techniques. AIBSNF seems to be ideally positioned to take advantage of the improvements in all these fields. It has the potential to impact a wide range of research and development activities in multiple industries. Due to the rapid pace of this development, numerous technological challenges exist. Identifying the right sensors, and mashing them up successfully at appropriate sample rates for time-synchronous data gathering, is possible but challenging. Data protection at each level of collection, use of the right algorithms, availability of computational power, and data science expertise are needed to successfully implement such technology on a commercial scale. The fields of sports and healthcare seem to be ideal areas where the proposed mashup technologies can be of significant benefit. AIBSNF provides a high-level understanding of how these technologies can be combined to develop applications in multiple fields. However, there still exist a whole host of technical challenges specific to the application domain. Further research and development are required for the successful application of AIBSNF in the highlighted industries.

## Data Availability

Materials required for the current review were previously published peer review articles which have been cited in the text and references.

## Abbreviations

BSN: Body sensor networks
AIBSNF: Artificial intelligence-based body sensor network framework
RTLS: Real-time location systems
AI/ML: Artificial intelligence and machine learning
ECG: Electrocardiogram
EMG: Electromyogram
ANN: Artificial neural networks
GSR: Galvanic skin resistance
LED: Light emitting diode
IMU: Inertial measurement units
LoS: Line of sight
NLoS: No line of sight
MLFF: Multi-level fusion framework
RA: Rheumatoid arthritis
TCM: Traditional Chinese medicine
